# 

**Published:** 1898-11

**Authors:** 


### NOTICES.

### To the Members of the Ohio State Dental Society.

BY GRANT MOLLYNEAUX, PRESIDENT.

*Dear Sir:*—Permit me to call your attention to the next annual meeting of the Ohio State Dental Society, to be held in Columbus on the first Tuesday, Wednesday and Thursday of December of this year. This meeting bids fair to be one of the best meetings in the history of the Society. We will have clinics, papers and exhibits of everything modern in dentistry. You cannot afford to be absent. Please cancel dates in your appointment book NOW, and make every effort to be with us from beginning to end. The dates are the 6th, 7th and 8th of December. Every dentist in the State who is interested in the advancement of his profession should unite with the representative body of the State and make his influence felt, and a power towards correcting the abuses now too prevalent amongst incompetent practitioners of dentistry. We can only accomplish good results by a united effort. Please lend us your aid at the next meeting. You will hear from us again, and a program will be sent you in due time; but remember the days of the next Ohio State Dental Society meeting, and make arrangements to be present.

